# Effects on blood pressure, cardiac mass and function after renal denervation in patients with resistant hypertension

**DOI:** 10.1186/1532-429X-18-S1-P106

**Published:** 2016-01-27

**Authors:** Enver Tahir, Lennart Well, Maxim Avanesov, Fabian J Brunner, Karsten Sydow, Gunnar Lund, Gerhard Adam, Andreas Koops

**Affiliations:** 1 grid.13648.380000000121803484Diagnostic and Interventional Radiology, University Hospital Eppendorf, Hamburg, Germany; 2 grid.13648.380000000121803484General and Interventional Cardiology, University Heart Center Hamburg, Hamburg, Germany

## Background

In recent years, catheter-based renal denervation (RDN) has been investigated as a promising strategy in the treatment of resistant hypertension. Due to a reduction of whole body sympathetic activity, additional benefits on cardiac adaptation have been proposed. The purpose of this study was to investigate the effect of RDN on blood pressure (BP) as well as cardiac mass and function via cardiac magnetic resonance imaging (CMRI).

## Methods

RDN was performed on 15 patients with a history of resistant hypertension (9 male and 6 female, mean age 67.2 years). Office and ambulatory long term blood pressures were measured before and 12 months after RDN. For quantitative CMRI, an electrocardiographically triggered steady-state free precession (SSFP) cine sequence (TR/TE, 3.2/1.6 ms; pixel-size, 1.7 mm × 1.7 mm) was performed in short- and long-axis views before and 12 months after RDN. Quantitative analysis included end-diastolic (EDV) and end-systolic volumes (ESV), stroke volume (SV), left ventricular ejection-fraction (EF) as well as end-diastolic (EDMM) and end-systolic myocardial mass (ESMM). CMRI data were analyzed by two independent observers using the HeAT-Software. Data are given as the mean of both observers. Statistical analysis was performed using GraphPad Prism 4 and Excel, Microsoft.

## Results

In patients with resistant hypertension, RDN let to a significant decrease of EDMM (162.8 ± 52.4 g vs 152.5 ± 52.0 g; p< 0.05) and ESMM (166.5 ± 54.5 g vs 154.9 ± 53.23 g; p< 0.05) within 12 months after intervention. EDV (163.0 ± 38.6 ml vs 168.1 ± 49.8 ml), ESV (70.4 ± 33.0 ml vs 72.0 ± 39.8 ml), SV (92.7 ± 20.7 ml vs 96.1 ± 30.6 ml) and EF (0.583 ± 0.116% vs 0.588 ± 0.126%) did not change on a significant level. BP measurements revealed a significant decrease of the minimal diastolic BP in ambulatory long term measurements (53.3 ± 9.2 mmHg vs 48.8 ± 12.8 mmHg; p< 0.05). No additional significant changes in average, systolic or diastolic, office or ambulatory, diurnal or nocturnal BP measurements was detected.

## Conclusions

RDN lead to a decrease of cardiac mass within 12 months after intervention without a significant change of left ventricular function. Additionally, minimal diastolic BP showed a significant decrease in ambulatory measurements, whereas no other significant changes in BP levels were detected. Despite a rather small effect on blood pressure, additional benefits of RDN regarding cardiac adaptation can be observed. Further investigations might lead to a specified selection of patients who benefit from RDN beyond reduction of BP.

